# A Terahertz Metasurface Sensor Based on Quasi-BIC for Detection of Additives in Infant Formula

**DOI:** 10.3390/nano14100883

**Published:** 2024-05-19

**Authors:** Mingjun Sun, Jie Lin, Ying Xue, Weijin Wang, Shengnan Shi, Shan Zhang, Yanpeng Shi

**Affiliations:** School of Integrated Circuits, Shandong University, Jinan 250100, China

**Keywords:** additives in infant formula, THz spectroscopy, split ring metasurface, BIC, fingerprint detection

## Abstract

Prohibited additives in infant formula severely affect the health of infants. Terahertz (THz) spectroscopy has enormous application potential in analyte detection due to its rich fingerprint information content. However, there is limited research on the mixtures of multiple analytes. In this study, we propose a split ring metasurface that supports magnetic dipole bound states in the continuum (BIC). By breaking the symmetry, quasi-BIC with a high quality (Q) factor can be generated. Utilizing an angle-scanning strategy, the frequency of the resonance dip can be shifted, resulting in the plotting of an envelope curve which can reflect the molecular fingerprint of the analytes. Two prohibited additives found in infant formula, melamine and vanillin, can be identified in different proportions. Furthermore, a metric similar to the resolution in chromatographic analysis is introduced and calculated to be 0.61, indicating that these two additives can be detected simultaneously. Our research provides a new solution for detecting additives in infant formula.

## 1. Introduction

Melamine is a chemical raw material that is not allowed to be used in food or related ingredients. In the past few years, melamine has been illegally added to infant formula to increase the apparent crude protein value, resulting in renal failure in infants [1]. In addition to melamine, excessive ingestion of vanillin can also affect the function of the kidney [2,3]. Therefore, ensuring infant health and safety poses a challenge in effectively detecting additives in infant formula. At present, various techniques have been employed for the analysis of these two additives, such as high-performance liquid chromatography [4], near-infrared spectroscopy [5], and solid-phase microextraction [6]. Apart from these methods, terahertz (THz) spectroscopy can also be used for the analysis.

THz spectroscopy ranges between microwave and far infrared, typically from 0.1 to 10 THz, containing information such as molecular vibration and rotation that exhibits fingerprint characteristics [7,8]. With technological advancements, THz spectroscopy is gradually being applied to different fields such as food product safety [9], biomedical research [10], and chemistry [11]. Moreover, THz spectroscopy enables non-destructive detection because of its low photon energy causing minimal damage to the analytes, which is also one of the promising aspects of THz spectroscopy technology [12]. Nevertheless, the mismatch between the THz wavelength in the micrometer scale and the absorption cross-section of analytes results in extremely weak interactions between THz waves and analytes, leading to very low sensitivity of detection [13]. This limitation hinders the development of THz spectroscopy technology in the field of trace analytes detection. Therefore, a large volume of analytes is usually required in fingerprint detection [14].

In past research, THz time-domain spectroscopy (THz-TDS) has been demonstrated as a reliable method for measuring the refractive index and absorption coefficient of analytes, but this method uses a huge system and requires large sample amounts [15,16]. To solve the problem, a THz fingerprint sensor based on hybrid-induced transparency was proposed, which observed molecular fingerprints through the coherent coupling of a broadband mode and a narrowband mode, thereby enhancing the sensitivity of the detection [17,18]. In addition, nano-antenna metamaterial and metallic grooves can also be used to detect analytes [19,20]. Although these methods enhance the sensitivity of detection, the use of metal materials causes inherent damping and their narrowband sensing enhancements limit the detection of broadband fingerprints. Recently, a new THz fingerprint sensor has been proposed where the structure interacts strongly with analytes at the resonance frequency, altering the transmittance or reflectance. By utilizing strategies such as angle scanning, tunable graphene, and micro-ribbons, the resonance frequency of the sensor can be shifted over a wide spectral range, ultimately reflecting the trend of variation in the extinction coefficient of the analytes [21,22,23,24,25]. Due to the strong interaction at the resonance frequency, there is a significant enhancement in the sensitivity of detection. However, these studies mainly focus on studying a single specific analyte, rather than a mixture of various analytes in practical applications.

In this paper, we propose a metasurface sensor that can sense mixtures of multiple analytes. The sensor is based on quasi-bound states in the continuum (quasi-BIC) that is derived from BIC. BIC is a strong interaction with an infinite Q factor and zero leakage [26]. While the electromagnetic wave with frequency coexists with an extended state in the radiating continuum, it remains perfectly localized without radiation [27]. Quasi-BIC with radiation leakage can be obtained via some physical changes to the structural parameters of the metasurface and can be tuned to a high Q factor with an ultra-narrow radiation linewidth [28]. The metasurface is composed of a periodic array of silicon (Si) split rings, where the resonance frequency can be tuned by changing the incidence angle of the wave, enabling broadband detection. This feature provides sufficient bandwidth for detecting mixtures. And at the resonance point, the electromagnetic field is concentrated at the aperture of the split ring, facilitating the interaction between analytes and the electromagnetic field, thereby enhancing detection sensitivity. Even trace amounts of analytes can be identified. Due to these two advantages, this design is applied to detect melamine and vanillin in infant formula. Furthermore, different proportions of melamine and vanillin are analyzed and a metric for assessing the distinguishability of these two additives is introduced. The split ring metasurface provides a reliable approach for the application of the THz fingerprint sensor in food safety.

## 2. Materials and Methods

**Figure 1:**
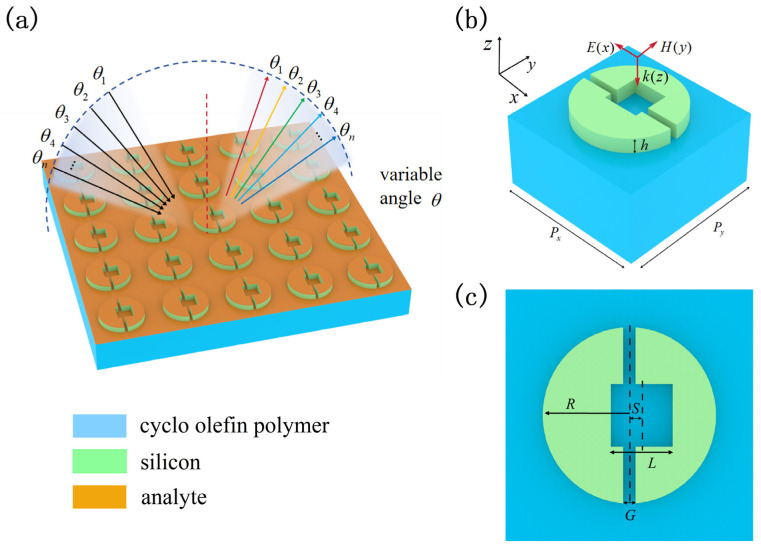
(**a**) Schematic diagram of the Si split ring metasurface which supports quasi-BIC. THz waves incident on the metasurface at different angles. (**b**) The unit cell of the metasurface. (**c**) The plan view of the unit cell.

Figure 1a shows the metasurface composed of a periodic array of Si split rings arranged in a square lattice. The orange thin layer structure is the analyte covering the metasurface. An x-polarized plane wave is normally incident on the metasurface. By varying the incident angle θ, an envelope curve of resonance dip transmittance can be formed, reflecting the extinction coefficient of the analytes. The geometric parameters of the metasurface are labeled in Figure 1b,c. The lattice constant of the Si split ring array is $P_{x}$ = $P_{y}$ = 78 µm. The array is positioned on a cyclo olefin polymer (COP) substrate known for its extremely low dielectric loss in the THz range, only 4.31 × 10^−4^, making it an excellent choice as a substrate material [29]. The unit cell of the split ring metasurface is composed of a cubic etched cylindrical structure, with an outer ring radius R = 33 µm, thickness h = 12 µm, and inner cubic etch dimensions L = 22 µm in length and width. The gap between the two half rings is G = 1.7 µm. To utilize the quasi-BIC generated by breaking the symmetry, the inner cubic etch needs to be shifted along the y-axis away from the center by S = 8 µm. The optical characteristics of the metasurface are simulated using 3D finite-difference time-domain (FDTD) solution software. Lumerical FDTD Solutions (2018), a commercial software developed by Lumerical (Canonsburg, PA, USA), is utilized for electromagnetic simulations. In the simulation, the perfectly matched layer (PML) is applied along the z-direction, and periodic boundary condition with phase correction is applied in the x- and y-direction. In practical realization, COP can achieve heterogeneous bonding with glass-like substrates through water vapor plasma treatment [30]. Therefore, it may be possible to create a thin layer of glass on the silicon surface, and then this glass layer is used to facilitate silicon production on COP. The Si split ring can be fabricated through mask photolithography and deep reactive ion etching [31]. Fifty unit cells can be used to compose a minimal sensor. The final sensor size is 3.9 mm [32].

## 3. Results and Discussion

**Figure 2:**
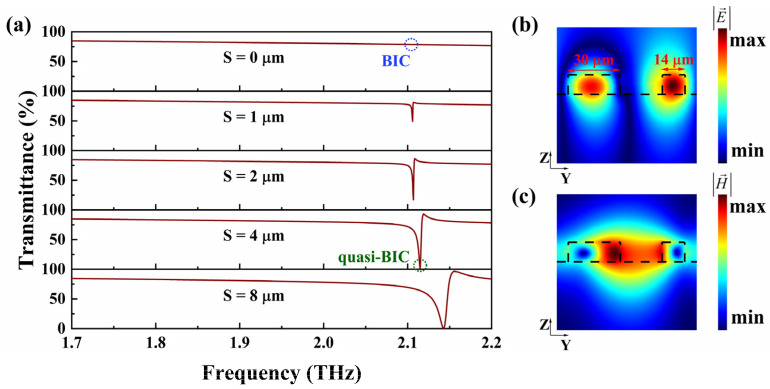
(**a**) Transmittance spectra of the metasurface with different S values. The blue circle is the BIC and the green circle is the quasi-BIC. (**b**) The absolute value distribution of the electric field when S is 8 μm and frequency is 2.14 THz. (**c**) The absolute value distribution of the magnetic field when S is 8 μm and frequency is 2.14 THz. The black lines represent the edges of the split ring.

Figure 2 illustrates how the asymmetrically distributed holes convert BIC into quasi-BIC as S increases when THz waves are vertically incident. It also shows the distribution of the electromagnetic field in the y-z plane at the resonance point. In Figure 2a, it can be observed that the resonance dip disappears when S = 0 μm. At the resonance frequency, the electromagnetic field is entirely confined to the metasurface, with no energy leaking from the resonance into free space, resulting in the disappearance of the transmittance dip due to an infinite high-quality (Q) factor [33,34,35,36]. As S increases from 0 to 8 μm, the transmittance dip gradually widens, and the Q factor decreases. Correspondingly, the BIC is transformed into a quasi-BIC. Figure 2b,c depict the electric and magnetic field distributions in the y-z plane of the metasurface. Strong electric fields with opposite directions are formed in the left and right rings and a magnetic dipole (MD) response is excited, while there is a strong magnetic field distribution in the cubic etch. A part of the analyte can be placed within this etch. Therefore, such an electromagnetic field distribution can enhance the interaction between analytes and the electromagnetic field, thereby improving the sensitivity of detection.

**Figure 3:**
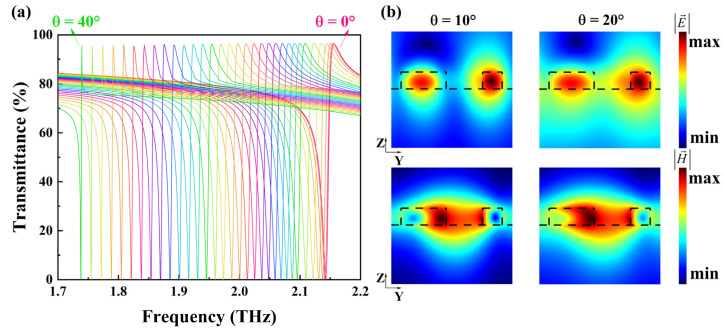
(**a**) Transmittance spectra of the metasurface for the incident angle changing from 0° to 40°. (**b**) The electromagnetic field distributions at the respective resonance frequencies for different angles of incidence.

Investigating the tuning effect of the incident angle θ is crucial for achieving broadband fingerprint detection. In Figure 3a, the impact of the incident angle θ on the transmittance spectra is depicted. For each incident angle θ, there is a unity transmittance. As the incident angle θ increases from 0° to 40°, the position of the resonance dip experiences a red-shift (from 2.14 to 1.74 THz). The step size is 2° for angles below 14° and 1° for angles above 14°. It is noteworthy that the increment in the frequency shift of the transmittance dip increases with the rising incident angle θ, which means that the resonance frequency can shift over a wide range. Although the incident angle θ changes, the quasi-BICs still maintain the high-Q characteristic, and the transmittance undergoes minimal changes. It can be observed that as the angle of incidence increases, the Q factor also gradually increases. Therefore, we ultimately chose S = 8 μm, at which point the Q factor of the transmittance dip is lower, making it easier to identify the minimum value of the transmittance under large-angle incidence. Ultimately, by extracting the frequency and the transmittance of the resonance dip, a flat envelope curve approaching zero can be plotted. This envelope curve can serve as the foundation for fingerprint detection. This tuning effect of the incident angle θ can be explained by the collective nature of the quasi-BIC. The metasurface can be modeled as an effective medium [32]. Different incident angles of THz waves impinging on the metasurface result in different phases, leading to different effective impedances of the metasurface and hence different frequencies of resonance dips. It can be seen from Figure 3b that the distributions of electric and magnetic fields in the y-z plane at the respective resonance frequencies for different incident angles are changed slightly. There are similar localized phenomena for the electromagnetic field. The quasi-BIC is still dominated by the MD response. Therefore, changing the incident angle θ only results in a red-shift of the resonance dip, without altering the minimum value.

**Figure 4:**
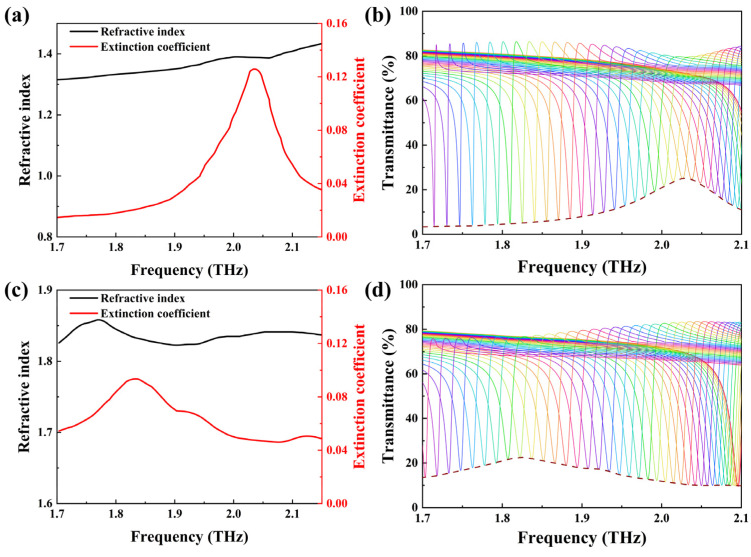
(**a**) The refractive index and extinction coefficient of melamine. (**b**) The transmittance spectra and their corresponding envelope curve for the metasurface with a 2 μm thick melamine layer deposition. (**c**) The refractive index and extinction coefficient of vanillin. (**d**) The transmittance spectra and their corresponding envelope curve for the metasurface with a 2 μm thick vanillin layer deposition.

Food safety is currently one of the international research hotspots, as excessive additives can cause irreversible harm to the human body. Vanillin and melamine are additives in milk powder that are strictly prohibited in excess. Particularly for infants, excessive absorption of these additives can lead to a variety of very serious diseases. Therefore, the focus is on a mixture composed of vanillin and melamine, achieving trace detection of these two additives. Molecular vibration and rotation lead to changes in the extinction coefficient, hence the extinction coefficient is used to reflect the trend of variations in the fingerprint. Since infant formula exhibits no absorption peaks within the range of 1.7 to 2.15 THz and has a relatively small extinction coefficient, attention is exclusively directed toward detecting these two additives [37]. Initially, the two additives are separated for detection to validate the accuracy of the THz metasurface sensor. The refractive index and extinction coefficient of these two additives can be obtained by measuring the samples using the THz-TDS system. Figure 4a depicts the refractive index (black) and extinction coefficient (red) curves of melamine within the frequency range of 1.7 to 2.15 THz [37]. These are extracted by measuring the transmittance spectra with the THz-TDS system and then calculating through the mathematical model for extracting optical parameters from the THz-TDS system, which is proposed by T.D.Dorney and L.D.Duvillaret and others [38,39,40]. The calculation formulas are as follows:

(1) $$n(\omega )=\frac{\phi (\omega )c}{\omega L}+1$$

(2) $$\kappa \left(\omega \right)=\frac{-ln\left\{\rho \left(\omega \right)\frac{{\left[n\left(\omega \right)+1\right]}^{2}}{4n\left(\omega \right)}\right\}c}{\omega L}$$

where L is the propagation length of the THz pulse, n(ω) is the refractive index, $\kappa \left(\omega \right)$ is the extinction coefficient, $\rho \left(\omega \right)$ is the ratio of the amplitude modulus of the reference signal $E_{ref}(\omega )$ and the sample signal $E_{sam}(\omega )$ after passing through a sample, and ϕ(ω) is the phase difference between $E_{ref}(\omega )$ and $E_{sam}(\omega )$. In Figure 4a, it can be seen that the refractive index n(ω) of melamine has a little variation with an average value of 1.35, while its extinction coefficient $\kappa \left(\omega \right)$ demonstrates remarkable change with a maximum value of 2.03 THz that corresponds to the fingerprint of melamine. Figure 4b shows the transmittance spectra after angle-scanning of the metasurface with a 2 µm thick melamine layer. The red curve is the envelope line extracted from the resonance dips. It can be observed that due to the optical loss of melamine, the envelope curve exhibits a maximum transmittance of about 25.2% at 2.03 THz, corresponding to the fingerprint of melamine. This validates the feasibility of our sensor for analyte detection. Figure 4c illustrates the refractive index and extinction coefficient of vanillin within the frequency range of 1.7 to 2.15 THz, also obtained using a THz-TDS system [41,42]. A characteristic absorption peak of vanillin is observed at 1.83 THz, with a maximum extinction coefficient of 0.094 and an average refractive index of approximately 1.84. The transmittance spectra after angle-scanning of the metasurface with a 2 µm thick melamine layer are shown in Figure 4d, with the envelope exhibiting a maximum transmittance of about 22.5% at 1.83 THz. The non-overlapping characteristic absorption peaks of these two additives provide the necessary conditions for the detection.

**Figure 5:**
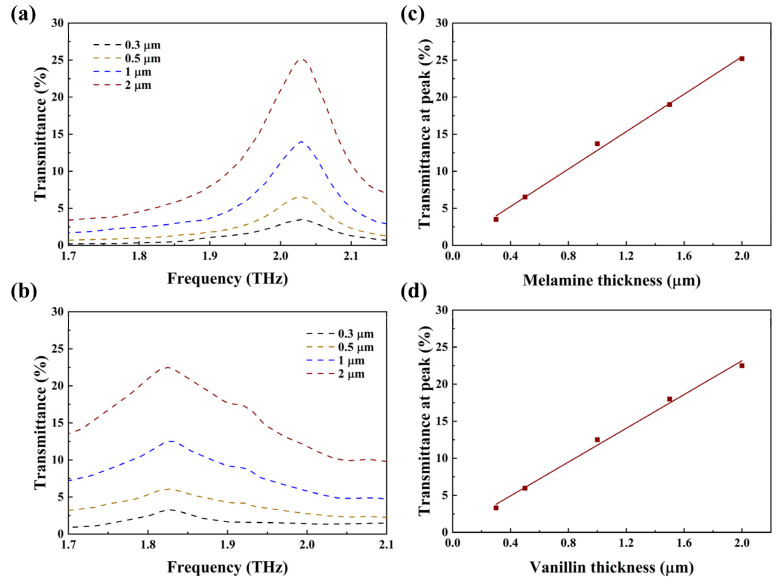
(**a**) The envelope curves of melamine with different thicknesses deposited on the metasurface. (**b**) The envelope curves of vanillin with different thicknesses deposited on the metasurface. (**c**) Transmittance at 2.03 THz versus the melamine thickness, and the red line is the fitted curve. (**d**) Transmittance at 1.83 THz versus the vanillin thickness, and the red line is the fitted curve.

To further evaluate the ability of fingerprint detection, the additives of different thicknesses are coated on the metasurface. Figure 5a,b depict the envelopes of melamine and vanillin deposited with thicknesses of 0.3 μm, 0.5 μm, 1 μm, and 2 μm, respectively. As the thickness increases, the transmittance at the peak of the envelope also increases. For melamine, this value increases from 3.5% to 25.2%. For vanillin, this value increases from 3.3% to 22.5%. Considering the signal-to-noise ratio of the actual THz-TDS system, the detection limits for vanillin and melamine are 52.8 μg cm^−2^ and 78.5 μg cm^−2^ [19]. Figure 5c,d show the transmittance values at their peaks for melamine and vanillin with the thickness ranging from 0.3 to 2 µm. It can be observed that the peak transmittance shows a good linear dependence on thickness. The red line is fitted through these five points, yielding the following equations: *y* = 0.126*x* + 0.002 (melamine) and *y* = 0.114*x* + 0.004 (vanillin). These two slope values can partially reflect the sensitivity of the detection. Different slopes of these two fitting curves stem from the different extinction coefficients of the two additives. Melamine exhibits a higher peak extinction coefficient compared to vanillin, so its corresponding fitting curve has a larger slope. The intercepts of these two curves are both close to zero, indicating that there are no absorption peaks in the absence of analytes. These findings demonstrate the ability to predict the thickness of the analyte by utilizing the transmittance at the peak of the envelope, enabling quantitative detections of melamine and vanillin.

**Figure 6:**
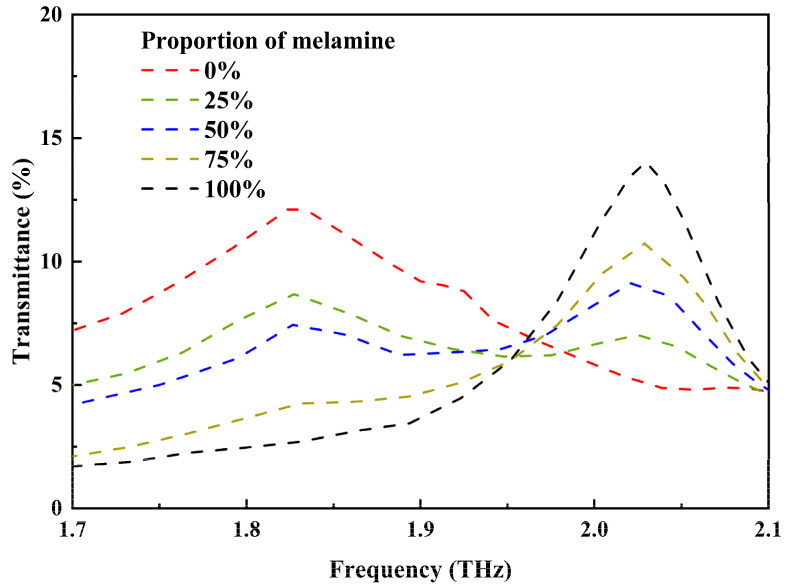
The envelope curves of 1 μm mixtures containing different proportions of melamine.

After detecting the characteristic absorption peaks of vanillin and melamine separately, a mixture of these two additives is analyzed. Figure 6 illustrates the envelope curves formed by the resonance dips of 1 μm mixtures containing different proportions of melamine. When the two additives are mixed in a 1:1 ratio, the envelope (blue curve) exhibits two distinct peaks, demonstrating the ability to distinguish between these two additives. Due to the higher peak extinction coefficient of melamine compared to vanillin, its absorption peak is more pronounced in Figure 6. As the proportion of melamine increases, its absorption peak becomes more pronounced. When the proportion reaches 75% (yellow curve), the absorption peak of vanillin almost disappears. This is because the positions of the two absorption peaks are close, and the trend of the melamine fingerprint masks that of vanillin. But the transmittance of the yellow curve near 1.83 THz remains higher than that of the black curve representing 100% melamine proportion. To quantify the distinguishability, a concept similar to the resolution in chromatographic analysis is introduced [43,44,45]. The resolution R is defined as follows:

(3) $$R=1.18\times \frac{f_{2}-f_{1}}{W_{h2}+W_{h1}}$$

where $f_{2}$ and $W_{h2}$ are the frequency and the peak width at half the height of the peak with higher frequency; $f_{1}$ and $W_{h1}$ are the frequency and the peak width at half the height of the peak with a lower frequency. The calculated R for the absorption peaks of the 1:1 mixture is approximately 0.61, indicating the degree of prominence in distinguishing between these two additives. In chromatographic analysis, when R is 0.6 and the integrated areas under the two chromatographic peaks are equal, the completely separated portion of the two peaks accounts for 88% and they can be observed. Therefore, the above study indicates that melamine and vanillin can be detected in the mixture at the same time.

## 4. Conclusions

In summary, a THz fingerprint sensor based on an angle-scanning strategy has been proposed, which is designed to detect two prohibited additives in infant formula. The metasurface is composed of a periodic array of Si split rings arranged in a square lattice, positioned on a COP substrate. When THz waves are vertically incident on the metasurface, MD quasi-BIC can be excited, forming a high-Q resonance. The electromagnetic field is confined to the metasurface, enhancing the sensitivity of detection. As the incident angle increases, the frequency of the resonance dip can be red-shifted, providing a wide bandwidth for detecting multiple analytes. By extracting the transmittance of the resonance dips, molecular fingerprint spectra of the analytes can be plotted. Two prohibited additives in infant formula, vanillin and melamine, are deposited on the metasurface. The absorption peaks of vanillin at 1.83 THz and melamine at 2.03 THz validate the accuracy of fingerprint detection. Subsequently, a mixture of the two additives is placed on the metasurface, and the two absorption peaks in the envelope confirm that these additives can be distinguished in the detection of infant formula. Furthermore, a concept similar to resolution in chromatographic analysis is introduced. It is calculated to be 0.61 for these two absorption peaks, which also proves that these two additives can be detected at the same time. This provides a measure for assessing whether the molecular fingerprints of the two analytes can be distinguished. Our sensor provides a powerful tool for detecting prohibited additives in infant formula, thereby promoting the advancement of food safety.

## Data Availability

Data underlying the results presented in this paper may be obtained from the authors upon reasonable request.
